# Rh-relaxin-2 attenuates degranulation of mast cells by inhibiting NF-κB through PI3K-AKT/TNFAIP3 pathway in an experimental germinal matrix hemorrhage rat model

**DOI:** 10.1186/s12974-020-01926-x

**Published:** 2020-08-28

**Authors:** Peng Li, Gang Zhao, Fanfan Chen, Yan Ding, Tianyi Wang, Shengpeng Liu, Weitian Lu, Weilin Xu, Jerry Flores, Umut Ocak, Tongyu Zhang, John H. Zhang, Jiping Tang

**Affiliations:** 1 Department of Physiology and Pharmacology, Basic Science, School of Medicine, Loma Linda University, Risley Hall, 11041 Campus St, Loma Linda, CA 92354 USA; 2 Department of Emergency Surgery, The Second Affiliated Hospital of Kunming Medical University, Kunming, 650101 China; 3 Traumatic Research Center of Yunnan Province, Kunming, 650101 China; 4 Department of Neurosurgery, Shenzhen Second People’s Hospital, The First Affiliated Hospital of Shenzhen University, Shenzhen, 518000 China; 5 Departments of Anesthesiology, Neurosurgery and Neurology, Loma Linda University School of Medicine, Loma Linda, CA 92354 USA

**Keywords:** Relaxin-2, Degranulation, Inflammation, Germinal matrix hemorrhage, Hydrocephalus, Mast cells

## Abstract

**Background:**

Mast cells play an important role in early immune reactions in the brain by degranulation and the consequent inflammatory response. Our aim of the study is to investigate the effects of rh-relaxin-2 on mast cells and the underlying mechanisms in a germinal matrix hemorrhage (GMH) rat model.

**Methods:**

One hundred seventy-three P7 rat pups were subjected to GMH by an intraparenchymal injection of bacterial collagenase. Clodronate liposome was administered through intracerebroventricular (i.c.v.) injections 24 h prior to GMH to inhibit microglia. Rh-relaxin-2 was administered intraperitoneally at 1 h and 13 h after GMH. Small interfering RNA of RXFP1 and PI3K inhibitor LY294002 were given by i.c.v. injection. Post-GMH evaluation included neurobehavioral function, Western blot analysis, immunofluorescence, Nissl staining, and toluidine blue staining.

**Results:**

Our results demonstrated that endogenous relaxin-2 was downregulated and that RXFP1 level peaked on the first day after GMH. Administration of rh-relaxin-2 improved neurological functions, attenuated degranulation of mast cells and neuroinflammation, and ameliorated post-hemorrhagic hydrocephalus (PHH) after GMH. These effects were associated with RXFP1 activation, increased expression of PI3K, phosphorylated AKT and TNFAIP3, and decreased levels of phosphorylated NF-κB, tryptase, chymase, IL-6, and TNF-α. However, knockdown of RXFP1 and PI3K inhibition abolished the protective effects of rh-relaxin-2.

**Conclusions:**

Our findings showed that rh-relaxin-2 attenuated degranulation of mast cells and neuroinflammation, improved neurological outcomes, and ameliorated hydrocephalus after GMH through RXFP1/PI3K-AKT/TNFAIP3/NF-κB signaling pathway.

## Background

GMH is the most common neurological disorder of newborns. It is defined as the rupture of immature blood vessels in the subependymal brain tissue of the premature infant [1]. The complications after GMH include neuroinflammation, hydrocephalus, primary and secondary brain injury, and developmental delay [2, 3]. Among all of those, the activation of inflammatory cascades could be the main factor leading to post-hemorrhagic consequences, such as long-term morphological and functional impairment [4]. Mast cells are considered the first responders and are able to initiate and magnify immune responses in the brain. Therefore, inhibition of the inflammatory response of mast cells is critically important at the early stage after GMH.

Mast cells are present in various areas of the brain and in the meninges [5]. Brain mast cells are mainly of a tryptase-chymase positive phenotype [6]. However, their number and distribution can quickly change in response to a number of environmental stimuli, such as trauma and stress. They release histamine, serotonim, tryptase, chymase, and TNF-α after activation. Furthermore, they can crosstalk indirectly with microglia in the release of cytokines. Therefore, treatments focused on reducing proinflammatory cytokines via inhibiting mast cells could be potentially important in attenuating mast cell degranulation and inflammation after GMH [7].

Relaxin-2 is a member of the insulin-like peptide family, which can bind to its receptor RXFP1 with high affinity. Several recent studies have reported that relaxin and RXFP1 are expressed in the local arteries of mice and rats [8–10]. In addition to a role in the reproductive system during pregnancy, a growing number of literature suggests that relaxin has extensive cardiovascular effects, such as protecting against fibrosis and early inflammation and promoting vasodilation and angiogenesis [11, 12]. Currently, a number of studies showed that PI3K-AKT is one of the downstream pathway of the interaction between relaxin and RXFP1 [13]. Moreover, tumor necrosis factor-alpha-induced protein 3 (TNFAIP3) plays an inhibitory role in terminating NF-κB signaling. However, it is unknown whether TNFAIP3 is a downstream mediator of relaxin-2 in exerting its stabilizing effect on mast cells after GMH.

Based on the abovementioned evidence, we hypothesized that rh-relaxin-2 treatment could suppress mast cell activation, consequently reduce the secretion of proinflammotory cytokines (Tryptase, chymase, IL-6, and IL-1β), improve neurological function in the short and long term and ameliorate PHH, and that these beneficial effects may be mediated by PI3K-AKT/TNFAIP3/NF-κB signaling (Supplementary Fig. 1).

## Materials and methods

### Animals

All experimental procedures were approved by the Institutional Animal Care and Use Committee at Loma Linda University. All studies were conducted in accordance with the United States Public Health Service’s Policy on Humane Care and Use of Laboratory Animals and reported according to the ARRIVE guidelines. One hundred seventy-three P7 Sprague-Dawley neonatal pups (weight = 12–14 g, Harlan, Livermore, CA) were randomly divided into Sham (*n* = 37) and GMH (*n* = 136) groups (Supplementary Table 1). All pups were housed with controlled temperature and 12-h-light/dark cycle and given ad libitum access to food and water. All rats (up to 21 days old) were returned to their home cages with mothers after doing surgery, drug administration, and behavior tests. Neither collagenase-induced GMH nor administration of rh-relaxin-2 caused mortality in this study. Investigators were blinded to the experimental groups when performing neurological tests, immunofluorescence, toluidine blue staining, and quantitation Western blot density.

### GMH model and experimental protocol

The procedure for the GMH model using collagenase infusion was performed as previously described [14]. Briefly, pups were anesthetized with isoflurane (3.0% induction, 1–1.5% maintenance) on a stereotaxic frame. After the skin was incised on the longitudinal plane and the bregma was exposed, a 27-gauge needle with 0.3 U clostridial collagenase (0.3 units of clostridial collagenase VII-S, Sigma-Aldrich, MO) was inserted at coordinates of 1.6 mmL, 1.5 mmA, and 2.7 mmV, and infused (1 μl/min) using a 10-μl Hamilton syringe (Hamilton Co, Reno, NV, USA) guided by a microinfusion pump (Harvard Apparatus, Holliston, MA). The needle was kept in place for an extra 10 min to avoid leakage and withdrawn at a speed of 0.5 mm/min. The pups were placed back onto a heated blanket after infusion and before being returned to their mothers, and euthanized at different time points according to the experimental design.

Intracerebroventricular drug administration was performed as previously described [15] as GMH induction, but the coordinates were at 1 mmA, 1 mmL, and 1.7 mmV.

Recombinant human relaxin-2 (rh-relaxin-2, Sigma-Adrich) was dissolved in phosphate-buffered saline (PBS). Pups were administered at different dosages (30 μg/kg, 60 μg/kg, and 90 μg/kg, GMH + rh-relaxin-2 groups) or PBS (GMH + vehicle group) via intraperitoneal injections at 1 h and 13 h after GMH. The Sham group was treated with the same volume of the solvent (PBS, 2 μl) of clostridial collagenase by stereotaxic injections as control.

Rat-derived RXFP1 siRNA (0.5 nm/2 μl, Thermo Fisher), scramble siRNA (0.5 nm/2 μl, Thermo Fisher), and clodronate liposome (15 μg/3 μl/rat, Encapsula Nano Sciences) were infused i.c.v. at 24 h prior to GMH induction.

Phosphatidylinsitol-3-kinase (PI3K) inhibitor LY294002 (50 mM, 2 μl, Sigma) was infused by i.c.v. injection at 1 h prior to GMH induction.

#### Experiment 1

The time course of endogenous relaxin-2, its receptor RXFP1, and the mast cell marker chymase and tryptase in the whole brain at 0.5, 1, 3, 5, and 7 days after GMH was analyzed by Western blot. The cellular localization of receptor RXFP1 and tumor necrosis factor-α-induced protein 3 (TNFAIP3) was detected at 1 day after GMH by double immunofluorescence staining on mast cells. Thirty-six rat whole brains were collected after perfusion with cold PBS at 0 (naive), 0.5, 1, 3, 5, and 7 days after GMH for Western blot (Supplementary Fig. 2).

#### Experiment 2

The outcome of rh-relaxin-2 treatment was assessed during the first 3 days and between 21 and 28 days after GMH. The pups were randomly divided into 5 groups: Sham, GMH + PBS, GMH + rh-relaxin-2 (30 μg/kg), GMH + rh-relaxin-2 (60 μg/kg), and GMH + rh-relaxin-2 (90 μg/kg). Exogenous rh-relaxin-2 (Sigma) was dissolved in PBS and administered in a total volume of 60 μl intraperitoneally (i.p.) at 1 h and 13 h after GMH. Short-term (negative geotaxis and body righting reflex) and long-term (rotarod test, foot fault, and water maze) neurological tests were examined during the first 3 days and between 21 and 28 days after GMH, respectively. The Nissl staining samples were also collected at 28 days after GMH (Supplementary Fig. 2).

#### Experiment 3

To evaluate the mast cell activation, the number of mast cells was quantified in the perihematoma area and thalamus on the first day after GMH by toluidine blue staining. Eighteen pups were divided into groups: Sham (*n* = 6), GMH + vehicle (*n* = 6), and GMH + rh-relaxin-2 (60 μg/kg, *n* = 6) (Supplementary Fig. 2).

#### Experiment 4

To evaluate the effect of RXFP1 in vivo on mast cell degranulation after administration of rh-relaxin-2 post-GMH, clodronate liposome was administered i.c.v. on the left side of the brain to inhibit the microglial activation at 24 h prior to GMH induction. Meanwhile, RXFP1 small interfering RNA (RXFP1 siRNA) and scramble siRNA (Scr siRNA) were also infused by i.c.v. injection on the right side of the brain. The whole brain samples were collected to conduct Western blot analysis on the first day post-GMH and after being perfused with cold PBS. The pups were randomly divided into six groups: Sham, GMH + vehicle, GMH + vehicle + clodronate liposome, GMH + clodronate liposome + rh-relaxin-2 (i.p. 60 μg/kg), GMH + clodronate liposome + rh-relaxin-2 (i.p. 60 μg/kg) + Scr siRNA, and GMH + clodronate liposome + rh-relaxin-2 (i.p. 60 μg/kg) + RXFP1 siRNA (Supplementary Fig. 2).

#### Experiment 5

To assess the role of PI3K-AKT pathway in vivo on mast cell degranulation after administration of rh-relaxin-2 post-GMH, clodronate liposome was administered i.c.v. on the left side of the brain to inhibit the microglial activation at 24 h prior to GMH induction. At the same time, LY294002 was administered by i.c.v. injection at 1 h on the left side of the brain prior to GMH induction. The whole brains were collected for Western blot on the first day post-GMH after being perfused with cold PBS. The pups were divided randomly into the following groups: Sham, GMH + vehicle, GMH + vehicle + clodronate liposome, GMH + clodronate liposome + rh-relaxin-2 (i.p. 60 μg/kg), GMH + clodronate liposome + rh-relaxin-2 (i.p. 60 μg/kg) + DMSO, and GMH + clodronate liposome + rh-relaxin-2 (i.p. 60 μg/kg) + LY294002 (Supplementary Fig. 2).

### Immunofluorescence

Double fluorescence staining was performed as described previously [16]. Sections were blocked with 5% donkey serum for 1 h and incubated at 4 °C overnight with primary antibodies: rabbit anti-RXFP1 (1:100, Biorbyt, orb157275), mouse anti-tryptase (1:200, Abcam, ab2378), mouse anti-chymase (1:200, Santa Cruz, sc-59586), and rabbit anti-TNFAIP3 (1:100, Lifespan, LS-C352948) followed by incubation with appropriate fluorescence-conjugated secondary antibodies for 2 h at room temperature. Negative control staining was performed by omitting the primary antibody. Fluorescence microscopy and LASX software were used to image the sections (Leica DMi8; Leica Microsystems, Wetzlar, Germany).

### Neurological examinations

Neurological tests were performed in a random and blinded setup as previously reported [17]. Short-term neurological tests, namely negative geotaxis and righting reflex, were conducted from day 1 to day 3 after GMH. Long-term neurological tests, including rotarod, foot fault, and water maze tests, were performed from day 21 to day 28 after GMH.

In detail, negative geotaxis was tested to record the duration of the pups to turn 90° and 180° when positioned head downward on a 45° inclined plane. The maximum recording time was 60 s (three trials/pup/day). For the righting reflex, the pups were placed on their backs on a horizontal plane, and the time needed for the pup to right itself in a prone position on its four paws was recorded. The maximum recording time was 20 s (three trials/pup/day).

A foot fault test was conducted and the total numbers of missteps were recorded. When the animal’s forelimb or hind limb fell into one of the grid openings, a foot fault was recorded. The maximum recording time was 60 s.

In a rotarod test, the animals were placed on a rotating wheel (Columbus Instruments) and tested at a starting speed of 5 rpm and 10 rpm with acceleration at 2 rpm per 5 s. The time latency for the animals to remain on the rotating wheel and the speed at which animals fell down from the rotarod were measured and averaged from 3 repeated trials.

The water maze test used a circular pool (diameter: 110 cm) filled with water at 24 ± 1^o^C. A transparent escape platform (diameter: 11 cm) was submerged 1 cm beneath the water and placed at a fixed position at the center of one of the quadrants. On day 6, a probe trial was performed to assess spatial memory retention. During this trial, animals were allowed to swim freely for 60 s, but no platform was present. Swim distance, escape latency, velocity, and the percentage of time in the target quadrant were digitally recorded and analyzed by a tracking software (Noldus Ethovision).

### Toluidine blue staining

Frozen sections were stained in toluidine blue working solution for 2–3 min. Sections were dehydrated quickly through 95% and 2 changes of 100% alcohol (10 dips in each since stain fades quickly in alcohol) after being washed in distilled water for three times. Finally, sections were cleared in xylene and covered with a resinous mounting medium. Mast cells were counted at perihematoma and thalamus areas for 3 sections per sample (*n* = 6/group).

### Nissl staining

Nissl staining was conducted and analyzed as previously described [18]. Brain sections were dehydrated in 95% and 70% ethanol for 2 min and then washed in distilled water for 2 min. Sections were stained with 0.5% cresyl violet (Sigma-Aldrich, USA) for 2 min and washed in distilled water for 10 s followed by dehydration with 100% ethanol and xylene for 2 min twice, respectively, before a coverslip with Permount was placed. Volumes were calculated as the average delineated area from 10 μm sections taken at ≈ 2.5 mm, 1.2 mm, 0.7 mm rostral, and 2.9 mm caudal of the bregma multiplied by the depth of the cerebroventricular system. ImageJ software was used to measure cortical thickness and white matter area in Nissl stained histological brain sections. These indexes were relative to the control group [18, 19]. Calculations were performed in a blinded fashion.

### Western blot

Brain tissues were collected and stored in a – 80 ^o^C freezer after being perfused with cold PBS (0.1 M, pH 7.4). Western blot was performed as described previously [14, 20]. After extraction of protein samples, protein quantification was performed using Lowry methodology (BioRad, USA). Each sample containing 50 μg of protein were separated by SDS-PAGE gel electrophoresis and then transferred onto nitrocellulose membranes. Membranes were blocked with 5% milk and incubated with the following primary antibodies overnight at 4 °C: rabbit anti-RXFP1 antibody (Biorbyt, USA, orb157275), rabbit anti-relaxin 2 antibody (Invitrogen, USA, PA5-76483), mouse anti-mast cell tryptase antibody (Abcam, USA, ab2378), rabbit anti-mast cell chymase antibody (Santa Cruz, USA, sc-59586), rabbit anti PI3K (CST, USA, #4249), rabbit anti-phospho-AKT (CST, USA, #9271 s), rabbit anti-AKT(CST, USA, #9272), rabbit anti-TNFAIP3 antibody (Lifespan, USA, LS-C352948), rabbit anti-NF-κB (Novusbio, USA, NBP1-87760), rabbit anti-phospho-NF-κB (CST, USA, #3033S), rabbit anti-IL-6 (Abcam, USA, ab9324), rabbit anti-TNF-α (Abcam, USA, ab9755), and goat anti-β-actin (Santa Cruz Biotechnology, USA, sc-1616). β-actin was used as the internal loading control. Membranes were then incubated with horseradish-peroxidase conjugated secondary antibodies for 1 h at room temperature. Membranes were probed with an ECL Plus chemiluminescence reagent kit (Amersham Biosciences, USA). The relative density of protein was analyzed by ImageJ software (ImageJ 1.5, NIH, USA).

### Statistical analysis

All data were presented as a mean ± SD. All analyses were performed using GraphPad Prism 6 (GraphPad software). Normal distribution was first confirmed using the Shapiro-Wilk normality test. For the data that passed the normality test, the statistical differences among groups were further analyzed using one-way ANOVA followed by Tukey’s multiple comparison post hoc analysis. For the data that failed the normality test, Kruskal-Wallis one-way ANOVA on ranks was used, followed by Tukey’s multiple comparison post hoc analysis. *P* < 0.05 was considered statistically significant.

## Results

### Endogenous relaxin-2 was downregulated and RXFP1 level peaked on the first day after GMH

**Fig. 1:**
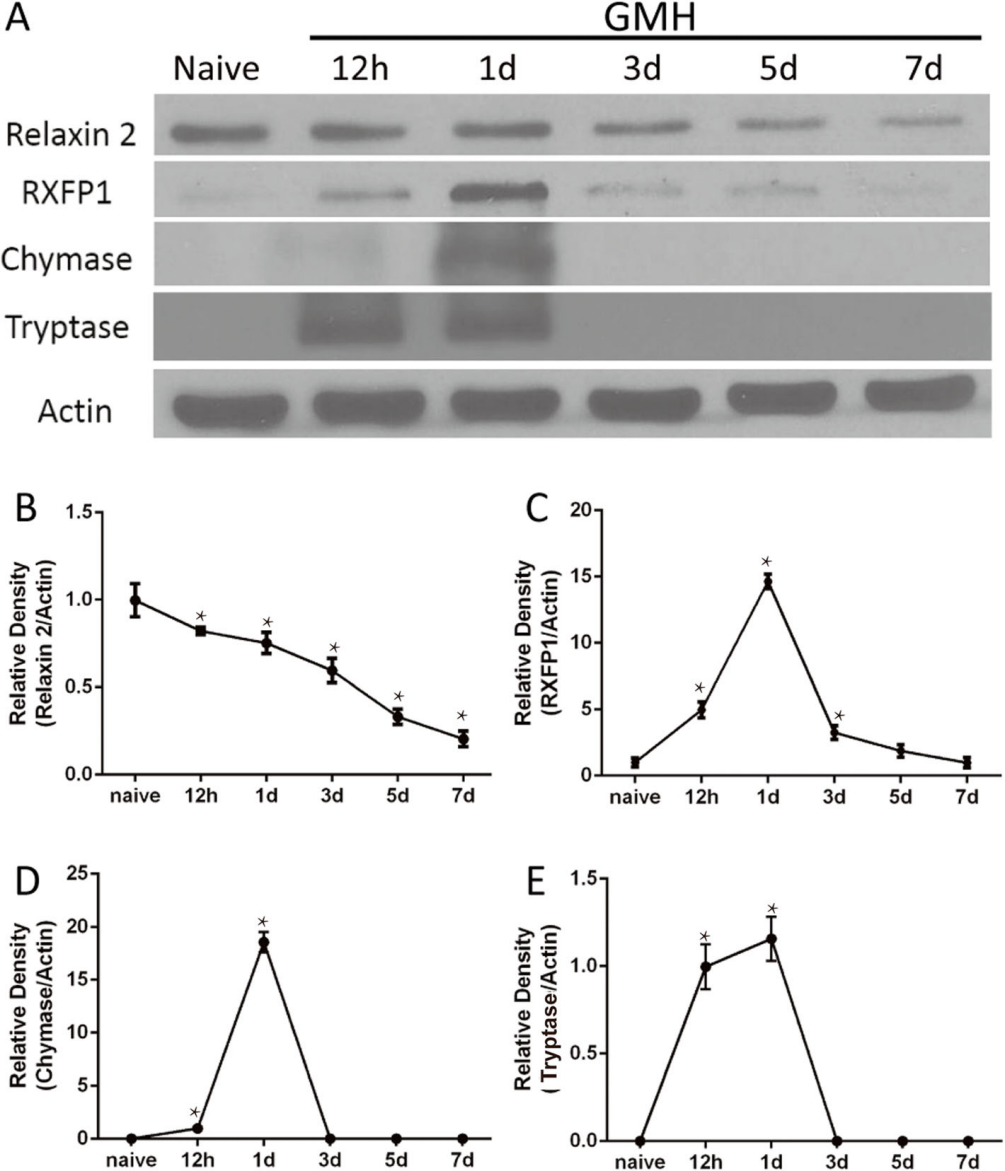
Time course of endogenous relaxin-2, RXFP1, chymase, and tryptase expression after GMH. Representative Western blot bands (**a**) and quantitative analysis of relaxin-2 (**b**), RXFP1 (**c**), chymase (**d**), and tryptase (**e**) expression in the whole brains after GMH. The relative density of each protein has been normalized against the Sham group. *n* = 6. *P < 0.05 vs Sham

**Fig. 2:**
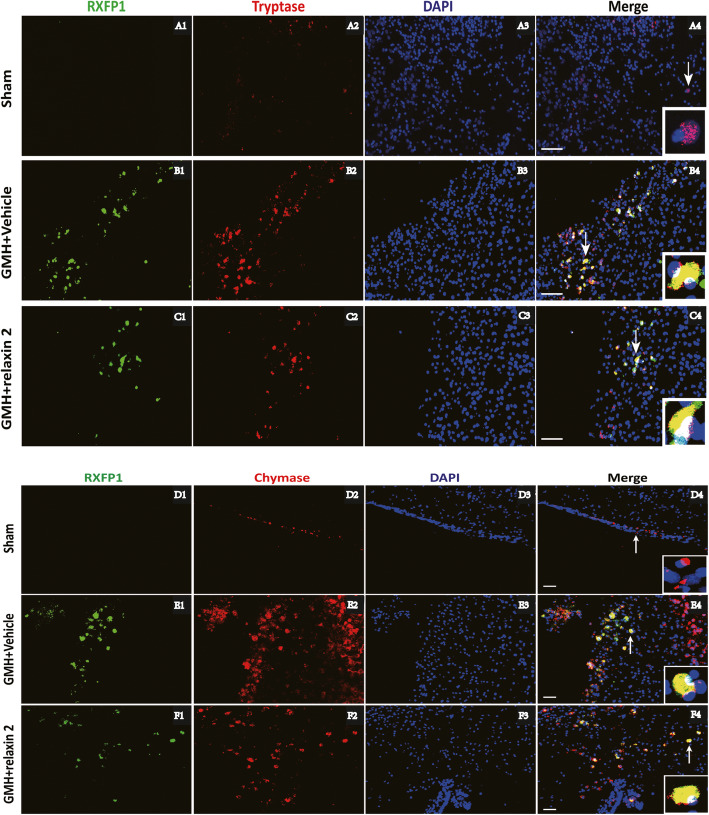
The cellular localization of RXFP1 in the perihematoma area of the brains. Representative images of double immunofluorescence staining showed that RXFP1 (A1, B1, and C1) was expressed on mast cells marked with tryptase (A2, B2, and C2) and chymase (D2, E2, and F2) on the first day after GMH. *n* = 6. Scale bar = 50 μm

Western blot results showed that there was a significant decrease in the expression of endogenous relaxin-2 at 12 h after GMH (Fig. 1a, b). The expression of RXFP1 increased at 12 h after GMH, peaked on the first day, and declined significantly on the third, fifth, and seventh day after GMH (Fig. 1a, c). Mast cell marker chymase expression increased and peaked on the first day and diminished on the third day after GMH (Fig. 1a, d). Additionally, tryptase, another marker of mast cells, increased dramatically at 12 h, peaked on the first day, and decreased on the third day after GMH (Fig. 1a–e). Double immunofluorescence staining demonstrated that the receptor RXFP1 was expressed abundantly on mast cells marked with tryptase (Fig. 2B2, B4, C2, C4) and chymase (Fig. 2E2, E4, F2, F4) on the first day after GMH. Furthermore, TNFAIP3 (Supplementary Fig. 3B and F) was also co-localized in mast cells marked by tryptase (Supplementary Fig. 3A and D) and chymase (Supplementary Fig. 3E and H) on the first day after GMH.

### rh-relaxin-2 treatment inhibited mast cell response after GMH

**Fig. 3:**
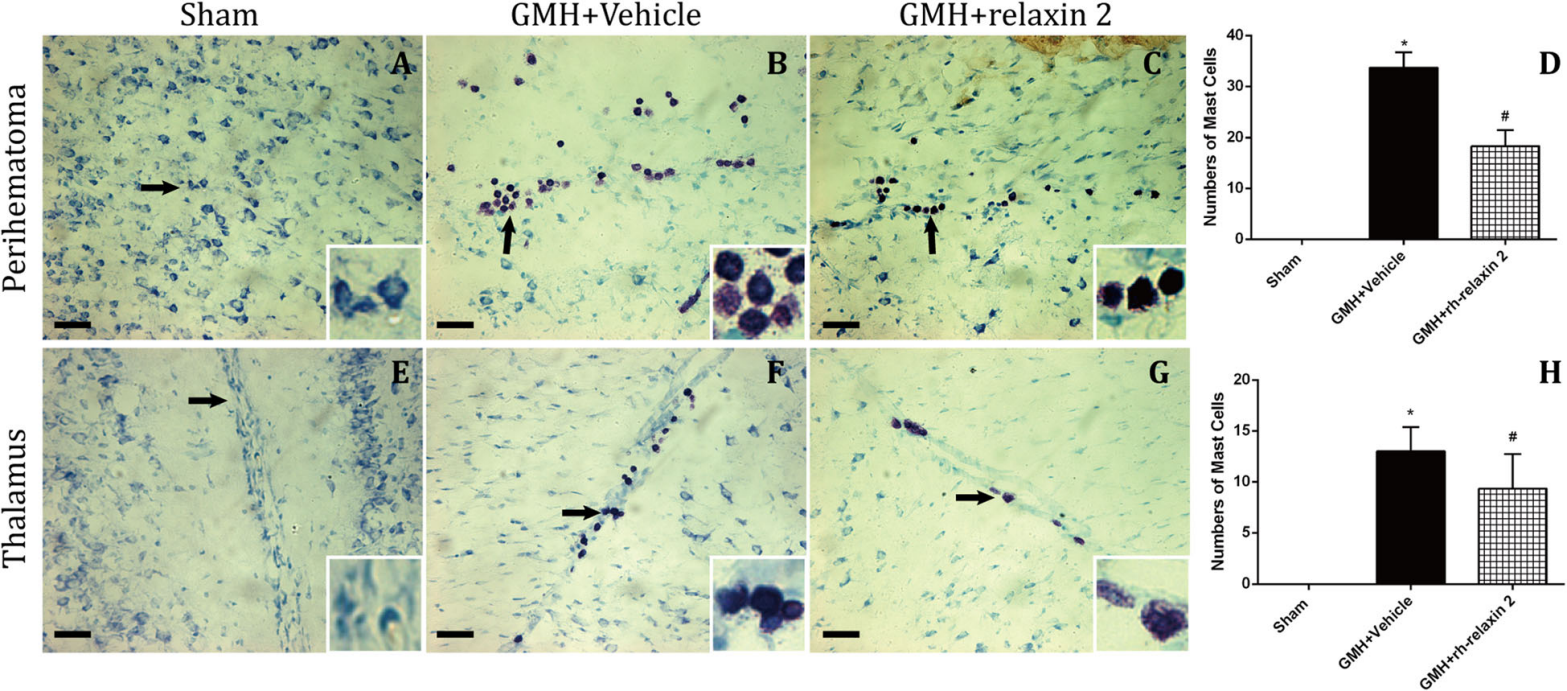
Toluidine blue staining of mast cells in the perihematoma area (**a**–**d**) and thalamus (**e**–**h**) and quantitation of mast cells on the first day after GMH. *n* = 6. Scale bar = 50 μm. *P < 0.05 vs Sham, #*P* < 0.05 vs GMH + vehicle

In order to explore whether rh-relaxin-2 inhibits mast cell degranulation after GMH, we used toluidine blue as the specific staining of mast cells in the perihematoma and thalamic areas and quantified the numbers of mast cells on the first day after GMH. The results showed that the total numbers of violet mast cells with rh-relaxin-2 treatment were decreased compared to the vehicle group in the perihematoma (Fig. 3b–d) and thalumic (Fig. 3f–h) areas. There were scarcely any violet mast cells in the sham groups (Fig. 3a, d, e, h).

### Intraperitoneal administration of rh-relaxin-2 improved short-term neurological function at 72 h post-GMH

**Fig. 4:**
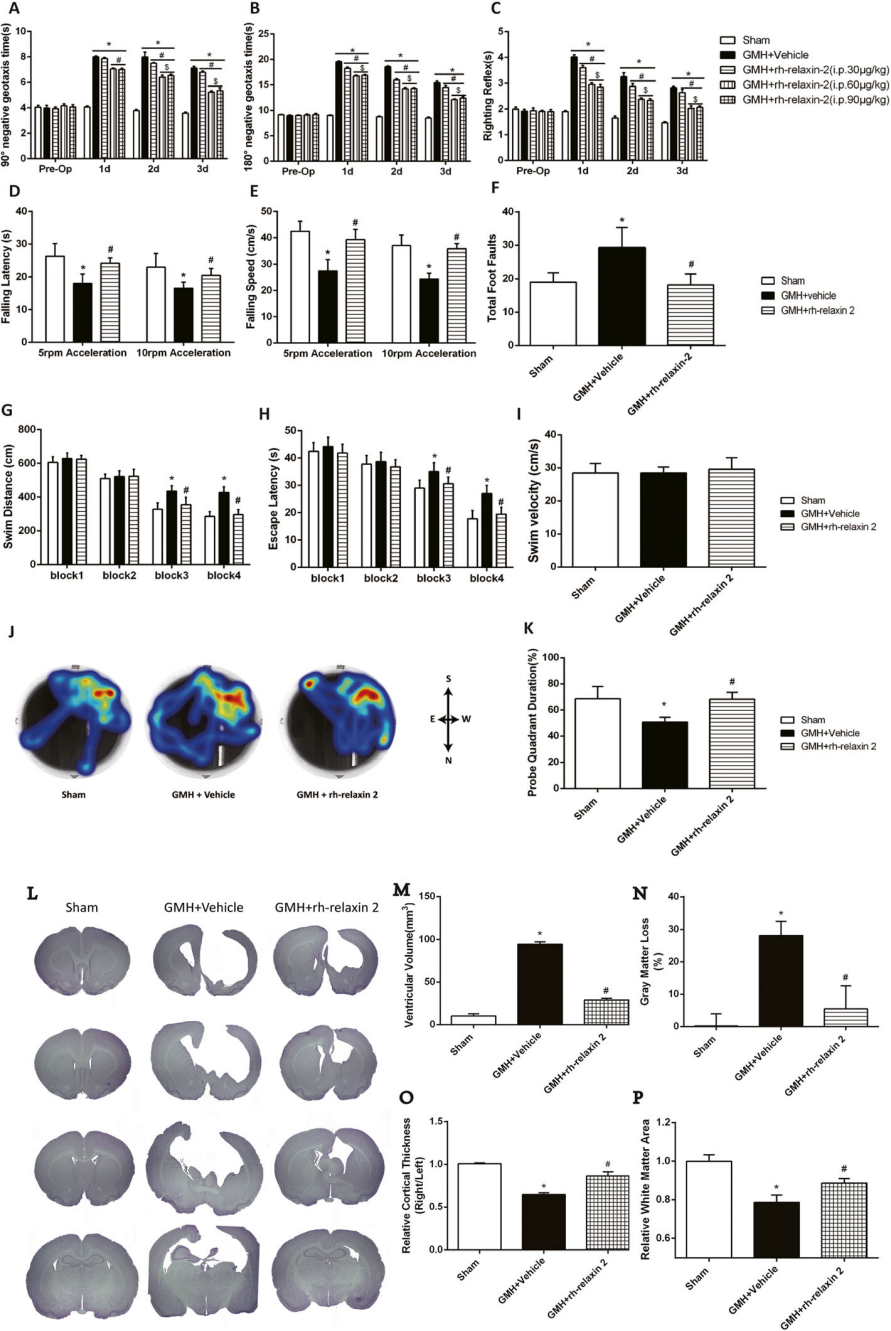
Intraperitoneal administration of rh-relaxin-2 improved short-term neurological function at 3 days after GMH. Negative geotaxis (**a**, **b**) and righting reflex (**c**) demonstrated that medium (60 μg/kg) and high (90 μg/kg) dosages of rh-relaxin-2 significantly improved neurological function compared to vehicle-treated pups in the first 3 days. *P < 0.05 vs Sham, #*P* < 0.05 vs GMH + vehicle, $*P* < 0.05 vs low dosage (30 μg/kg) of rh-relaxin-2, one-way ANOVA, Tukey’s test, *n* = 7/group. rh-relaxin-2 (60 μg/kg) treatment significantly increased the falling speed and falling latency in both 5 rpm - and 10 rpm (**d**, **e**) acceleration groups. In the foot fault test, animals in the vehicle group had significantly more total foot slips compared to the rh-relaxin-2 (60 μg/kg) treatment group (**f**). The water maze test showed that animals from the vehicle group swam significantly longer in 1 min (**g**), took more time to find the platform (**h**), and spent less time in the target quadrant during the probe trial (**j**, **k**) compared to the sham animals. In contrast, rh-relaxin-2-treated animals performed better (**j**, **k**) than vehicle. *P < 0.05 vs Sham, #*P* < 0.05 vs GMH + vehicle, one-way ANOVA, Tukey’s test, *n* = 10/group. In addition, rh-relaxin-2 administration reduced ventricular volume (**l**, **m**) and gray matter loss (**n**), and increased relative cortical thickness (**o**) and relative white matter area (**p**) significantly. *P < 0.05 vs Sham, #*P* < 0.05 vs GMH + vehicle, one-way ANOVA, Tukey’s test, *n* = 6

Three dosages (30 μg/kg, 60 μg/kg, and 90 μg/kg) of rh-relaxin-2 were administered via intraperitoneal injections at 1 h and 13 h after GMH. Pups in the vehicle group took significantly longer time to finish the action from head downward to the prone 90° (Fig. 4a) and 180° (Fig. 4b) position compared to the sham group in the first 3 days after GMH. There were significant differences in negative geotaxis between the three treatment groups and vehicle on the first, second, and third day after GMH. Among these treatment groups, both the medium and high dosages of rh-relaxin-2 improved short-term neurological function in negative geotaxis and body righting reflex (Fig. 4c). Considering the drug safety profile, we chose the medium dose of rh-relaxin-2 (60 μg/kg) for the following studies.

### rh-relaxin-2 treatment ameliorated long-term neurological deficits post-GMH

In the rotarod test, rh-relaxin-2 (60 μg/kg) treatment significantly increased the falling speed and falling latency in both 5 rpm (Fig. 4d, e) and 10 rpm (Fig. 4d, e) acceleration groups compared to the vehicle group. In the foot fault test, animals in the vehicle group had significantly more total foot slips compared to the rh-relaxin-2 (60 μg/kg)-treated group (Fig. 4f). Moreover, the water maze test showed that animals from the vehicle group swam a significantly longer distance (Fig. 4g), took more time to find the platform (Fig. 4h), and spent less time in the target quadrant during the probe trial (Fig. 4j, k) compared to sham animals. In contrast, rh-relaxin-2-treated animals performed better (Fig. 4j, k) than vehicle-treated animals. These findings indicated that rh-relaxin-2 treatment improved memory function at 28 days after GMH. There was no significant difference in swimming velocity among these 3 groups (Fig. 4i), meaning that the differences in finding the platform was related to the memory recovery, rather than the swimming ability.

### rh-relaxin-2 treatment attenuated ventricular dilation and gray matter loss and restored cortical thickness and white matter area after GMH

Ventricular dilation is a major demonstration of PHH. We evaluated whether this could be attenuated by rh-relaxin-2 treatment. Significant ventricular dilation (Fig. 4l) was observed in vehicle-treated animals, but the ventricular volume was reduced significantly with rh-relaxin-2 treatment (Fig. 4l, m). Gray matter loss was significant in vehicle-treated animals, while it was also significantly attenuated with rh-relaxin-2 treatment (Fig. 4l, n). Loss of cortical tissues was significantly attenuated with rh-relaxin-2 treatment (Fig. 4l, o) compared to vehicle-treated animals. Relative white matter area was significantly less in the vehicle group than that of the sham group and rh-relaxin-2 treatment pups (Fig. 4l, p).

### Knockdown of RXFP1 abolished the stabilizing effects of rh-relaxin-2 on mast cells after GMH

**Fig. 5:**
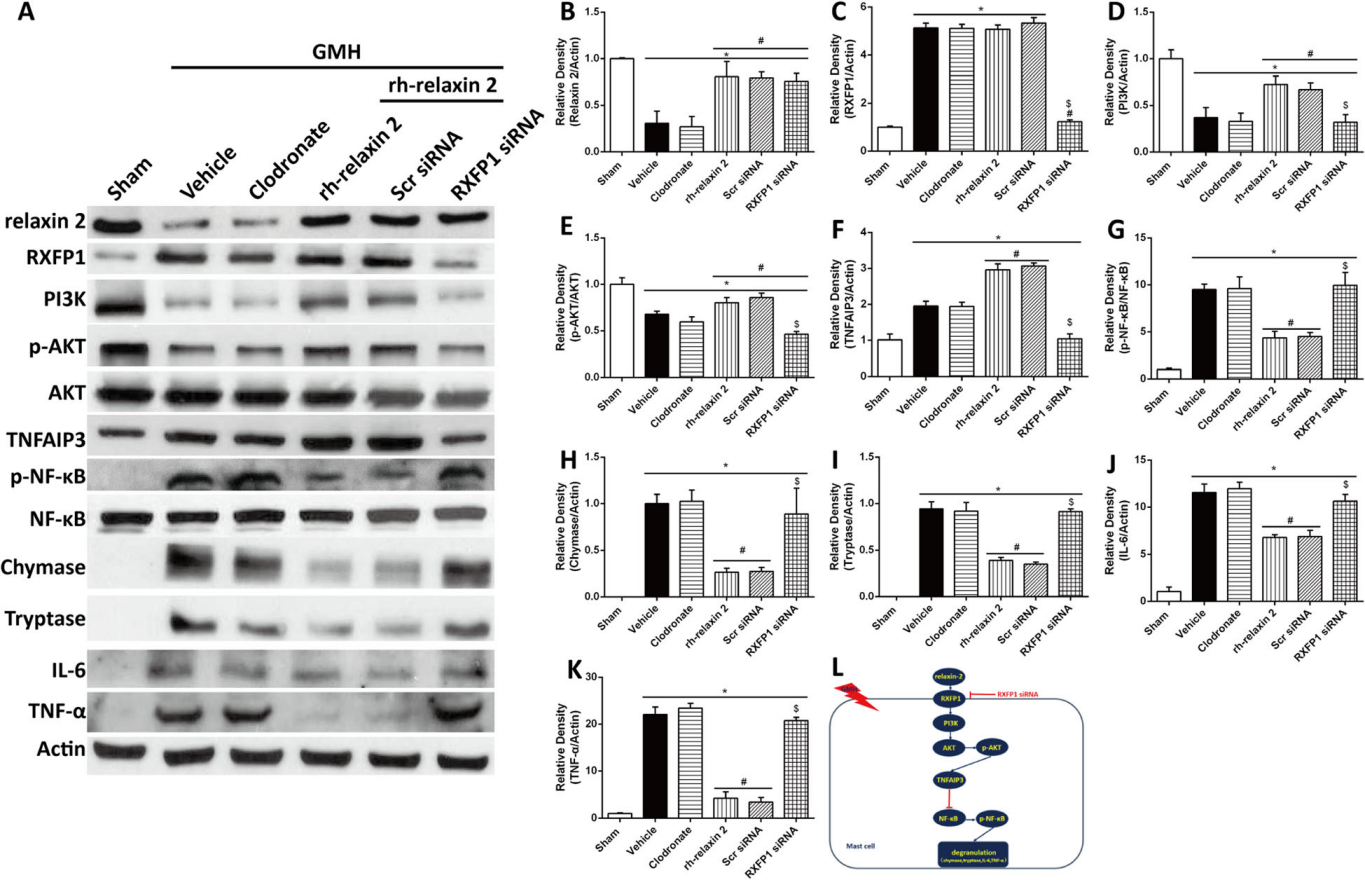
Knockdown of RXFP1 (**a**, **c**, **l**) by specific siRNA significantly inhibited the expression of PI3K (**a**, **d**), phosphorylated Akt (**a**, **e**), and TNFAIP3 (**a**, **f**) on the first day after intracerebroventricular injections. However, the expression of phosphorylated NF-κB (**a**, **g**) and inflammatory factors chymase (**a**, **h**), tryptase (**a**, **i**), IL-6 (**a**, **j**), and TNF-α (**a**, **k**) increased on the first day after GMH. *P < 0.05, Sham vs. RXFP1 SiRNA, #*P* < 0.05, GMH + vehicle vs. rh-relaxin-2, $*P* < 0.05, Scr SiRNA vs. RXFP1 SiRNA, one-way ANOVA, Tukey’s test, *n* = 6

Clodronate liposome was administered i.c.v. at 24 h prior to GMH to avoid the interference in immune response from macrophages or microglia (Fig. 5a, l, Supplementary figure 4). Western blot results showed that the expression of RXFP1 was increased dramatically after GMH compared to sham animals (Fig. 5a, c). Knockdown of RXFP1 by specific siRNA significantly inhibited the expression of RXFP1 in RXFP1 siRNA pups (Fig. 5a, c), PI3K (Fig. 5a, d), phosphorylated Akt (Fig. 5a, e), and TNFAIP3 (Fig. 5a, f), which was accompanied by the increase of phosphorylated NF-κB (Fig. 5a, g), chymase (Fig. 5a, h), tryptase (Fig. 5a, i), IL-6 (Fig. 5a, j), and TNF-α(Fig. 5a, k) expression on the first day after GMH.

### Inhibition of PI3K-Akt axis reversed the stabilizing effect of rh-relaxin-2 on mast cells after GMH

**Fig. 6:**
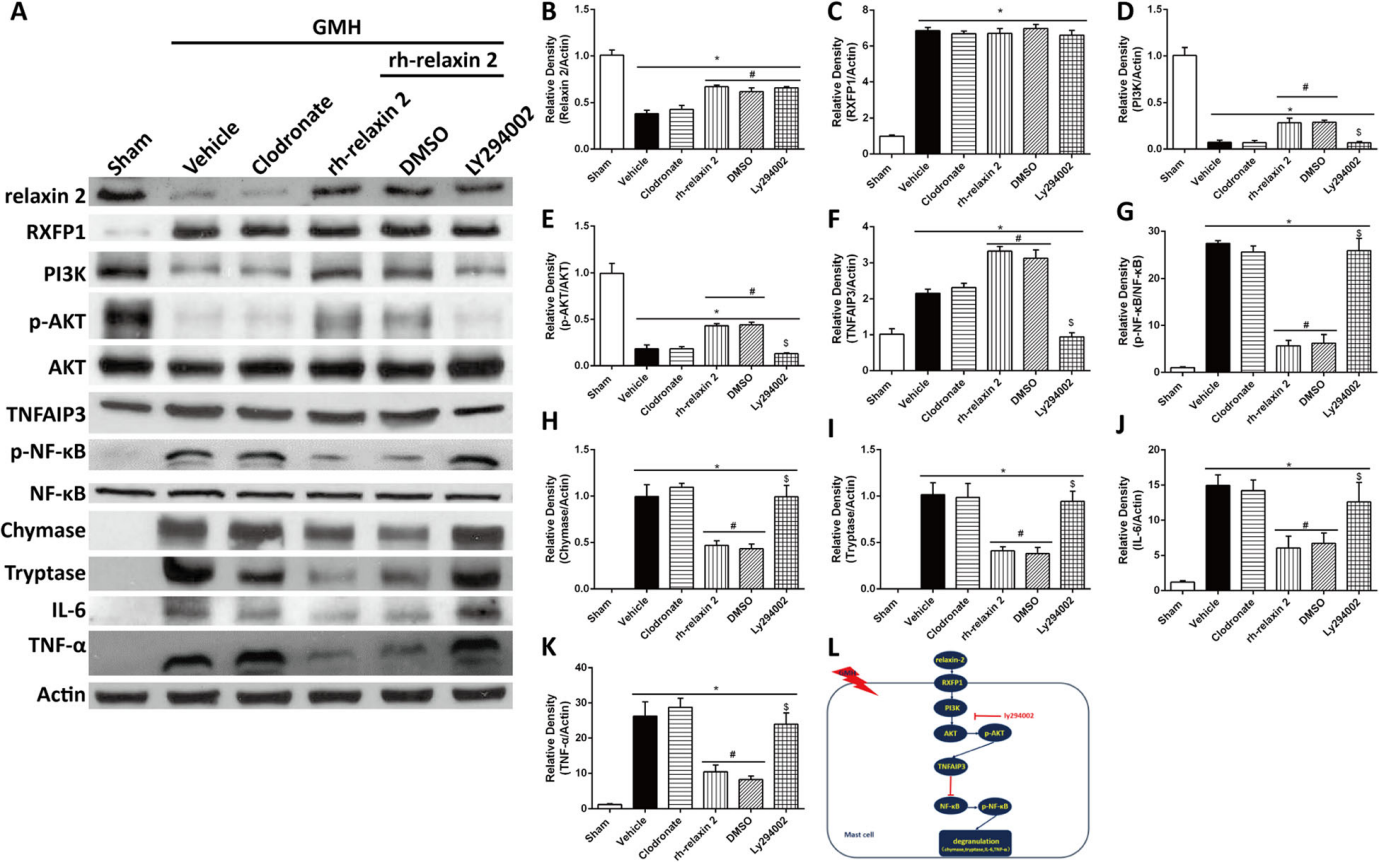
LY294002 significantly decreased PI3K (**a**, **d**, **l**), phosphorylated Akt (**a**, **e**), and TNFAIP3 (**a**, **f**) expression, which was accompanied by the increase of phosphorylated NF-κB (**a**, **g**), chymase (**a**, **h**), tryptase (**a**, **i**), IL-6 (**a**, **j**), and TNF-α (**a**, **k**) on the first day after GMH. *P < 0.05, Sham vs. LY294002; #*P* > 0.05, GMH + vehicle vs. LY294002; $*P* < 0.05, DMSO vs. LY294002, one-way ANOVA, Tukey’s test, *n* = 6

LY294002 was used to inhibit PI3K (Fig. 6a, l). The results demonstrated that PI3K (Fig. 6a, d) was reduced significantly by LY294002 on the first day after intracerebroventricular injection. The expression of phosphorylated AKT (Fig. 6a, e) and TNFAIP3 (Fig. 6a, f) also decreased subsequently. However, phosphorylated NF-κB (Fig. 6a, g), chymase (Fig. 6a, h), tryptase (Fig. 6a, i), IL-6 (Fig. 6a, j), and TNF-α (Fig. 6a, k) expression elevated significantly on the first day after GMH.

## Discussion

GMH is the most common neurological disorder of newborns, and the major neurological complications following intraventricular hemorrhage are neuroinflammation, cerebral palsy, PHH, and cognitive deficits [21]. Neuroinflammation is a trigger of secondary injury after GMH. Mast cells are the first responders in neuroinflammation after intracranial hemorrhage, which can release inflammatory mediators, such as cytokines, proteinases, and reactive oxygen species, to initiate and magnify the immune response in the brain. Therefore, inhibition of neuroinflammation focused on attenuating mast cell degranulation was our primary strategy to treat GMH in this study. Thus, we explored the therapeutic effects of rh-relaxin-2 in inhibiting the degranulation of mast cells and uncovered the potential mechanisms after GMH. Firstly, we observed that the expression of endogenous relaxin-2 decreased continuously and that its receptor RXFP1 increased on the first day but decreased in the late phase after GMH. The receptor RXFP1 was expressed abundantly after GMH on mast cells, which was marked by tryptase and chymase. Additionally, administration of rh-relaxin-2 at the dosage of 60 μg/kg improved short-term neurological functions in the first 3 days, and inhibited mast cell degranulation on the first day in the perihematoma and thamalus areas. It also attenuated the motor and memory dysfunction and reduced the ventricular dilation in the long-term studies. Moreover, knockdown of RXFP1 using RXFP1 siRNA aggravated mast cell degranulation and neuroinflammation, as shown by the decreased levels of PI3K, phosphorylated AKT, and TNFAIP3 and increase in chymase, tryptase, IL-6, and TNF-α. Furthermore, degranulation of mast cells and neuroinflammation were exacerbated with the inhibition of PI3K, which was concomitant with downregulation of phosphorylated AKT, TNFAIP3, and upregulation of chymase, tryptase, IL-6, and TNF-α.

The naturally circulating hormone relaxin-2 is a member of the insulin-like peptide family. It is well known to be used in cervical ripening, scleroderma, or systemic sclerosis and heart failure in basic and clinical research, due to its vasodilatory and organ protective actions. In this study, rh-relaxin-2 has been shown as a beneficial factor involved in attenuating the degranulation of mast cells. We observed that the endogenous relaxin2 decreased but its receptor RXFP1 increased as early as 12 h post-GMH, indicating a fast protective reaction to attenuate mast cell degranulation. This data was different from other research that RXFP1 mRNA was significantly downregulated on day 3 in a subarachnoid hemorrhage model of rabbit [22]. It might be because we chose an earlier time point at 12 h and 1 day post-GMH, which was concomitant with mast cell activation. Our further study showed that rh-relaxin-2 attenuated neurological deficits significantly in the short and long term after GMH. Therefore, improved outcomes were attributed to suppressed degranulation of mast cells by the systemic administration of rh-relaxin-2.

The exact mechanism by which rh-relaxin-2 exerts its protective effect in GMH still remains unclear so far. The 72-h intravenous administration of rh-relaxin-2 in acute myocardial infarction resulted in early beneficial effects, including reduced inflammation [10]. In our results, after knockdown RXFP1 by specific RXFP1 siRNA, mast cell markers chymase and tryptase, inflammatory cytokines IL-6 and TNF-α, and classic phosphorylated NF-κB all increased, which was consistent with the acute myocardial infarction outcomes. Hence, RXFP1 could be an important therapeutic target to reduce the degranulation of mast cells after GMH.

Our current results indicated that mast cell degranulation was exacerbated with inhibition of PI3K and also led to the decrease of phosphorylated AKT on the first day after GMH. Some research evidence demonstrated the PI3K-AKT axis as an important therapeutic target in attenuating degranulation of mast cells via suppressing immune responses, which has been validated as a major downstream pathway of RXFP1 activation [13]. RXFP1, as the receptor of relaxin-2, is expressed on mast cells abundantly after GMH. All of the abovementioned evidence supports our results observed in GMH.

It is known that TNFAIP3 is an endogenous anti-inflammatory factor that can reduce the expression of IL-6 and TNF-α by inhibiting NF-κB activation [23]. As shown in our results, TNFAIP3 expression was significantly reduced after knockdown of RXFP1 and PI3K in GMH animals. Thus, we hypothesized that TNFAIP3 may be a downstream factor of RXFP1 and the PI3K-AKT axis in the context of GMH. Meanwhile, the decrease of TNFAIP3 after inhibition by specific siRNA promoted the expression of phosphorylated NF-κB and inflammatory cytokines on the first day post-GMH. In this process, after activation of RXFP1 by rh-relaxin-2, an increase of TNFAIP3 mediated the reduction of NF-κB and functioned as a negative regulator of NF-κB. Previous reports also showed that IL-6 and TNF-α levels increased in TNFAIP3^-/-^ sham groups of an intracerebral hemorrhage mouse model, suggesting that TNFAIP3 deficiency caused spontaneous inflammation in the mouse brain, which was consistent with our results in GMH pups [24, 25]. Thus, our results, coupled with the previous research, suggested that rh-relaxin-2 attenuated GMH-induced inflammation through the PI3K-AKT/TNFAIP3/NF-κB pathway in mast cells.

There are some limitations in our current research. We only studied the mast cell activation rather than the detailed interaction between microglia and mast cells after GMH. In addition, we did not further explore the potential protective effects of rh-relaxin-2 on neurons and the reduction of glial scar in tissues in GMH.

## Conclusions

The activation of RXFP1 by rh-relaxin-2 could attenuate degranulation of mast cells and improve neurological function by inhibiting NF-κB through the PI3K-AKT/TNFAIP3 signaling pathway after GMH in a rat model. Therefore, rh-relaxin-2 may serve as a promising therapeutic agent to reduce neuroinflammation and secondary brain injury in GMH patients.

## Supplementary information

**Additional file 1:** **Figure S1.** Proposed mechanism. **Figure S2.** Experimental design. **Figure S3.** The cellular localization of TNFAIP3 in the perihematoma area of the brains. Representative images of double immunofluorescence staining showed that TNFAIP3 (B and F) was expressed on mast cells marked with tryptase (A) and chymase (E) on the first day after GMH. n = 6. Scale bar = 50 μm. **Figure S4.** Clodronate liposome inhibited the response of microglia in a GMH rat model. Representative images of immunofluorescence staining showed the expression of Iba1 in clodronate liposome + GMH animals (B, H) was inhibited compared to PBS + GMH animals (C, I) on the first day after GMH. n = 3. Scale bar = 50 μm. **Table S1.** Animal use in each experimental group.

## Data Availability

The datasets used and/or analyzed during the current study are available from the corresponding author on reasonable request.

## Abbreviations

GMH: Germinal matrix hemorrhage
PI3K: Phosphoinositide 3-kinase
TNFAIP3: TNF-alpha-induced protein 3
I.C.V.: Intracerebroventricular
IL-6: Interleukin 6
TNF-α: Tumor necrosis factor-alpha
IL-1β: Interleukin 1 beta
PHH: Post-hemorrhagic hydrocephalus
