# Supplementary material for: Rh-relaxin-2 attenuates degranulation of mast cells by inhibiting NF-κB through PI3K-AKT/TNFAIP3 pathway in an experimental germinal matrix hemorrhage rat model
Source: J Neuroinflammation. 2020 Aug 28;17:250. doi: 10.1186/s12974-020-01926-x (PMC7455905; doi:10.1186/s12974-020-01926-x)
Supplement: Supplementary file 1 — Additional file 1: Figure S1. Proposed mechanism. Figure S2. Experimental design. Figure S3. The cellular localization of TNFAIP3 in the perihematoma area of the brains. Representative images of double immunofluorescence staining showed that TNFAIP3 (B and F) was expressed on mast cells marked with tryptase (A) and chymase (E) on the first day after GMH. n = 6. Scale bar = 50 μm. Figure S4. Clodronate liposome inhibited the response of microglia in a GMH rat model. Representative images of immunofluorescence staining showed the expression of Iba1 in clodronate liposome + GMH animals (B, H) was inhibited compared to PBS + GMH animals (C, I) on the first day after GMH. n = 3. Scale bar = 50 μm. Table S1. Animal use in each experimental group. [file 12974_2020_1926_MOESM1_ESM.pdf]

Rh-relaxin-2 Attenuates Degranulation of Mast Cells by Inhibiting NF-κB through PI3K-AKT/TNFAIP3 Pathway in An Experimental Germinal Matrix Hemorrhage Rat Model

Peng Li[a, †], Gang Zhao [a, b, c, †], Fanfan Chen[d, †], Yan Ding[a], Tianyi Wang[a], Shengpeng Liu[a], Weitian Lu[a], Weilin Xu[a], Jerry Flores[a], Umut Ocak[a], Tongyu Zhang[a], John H. Zhang[a, e], Jiping Tang[a, *]

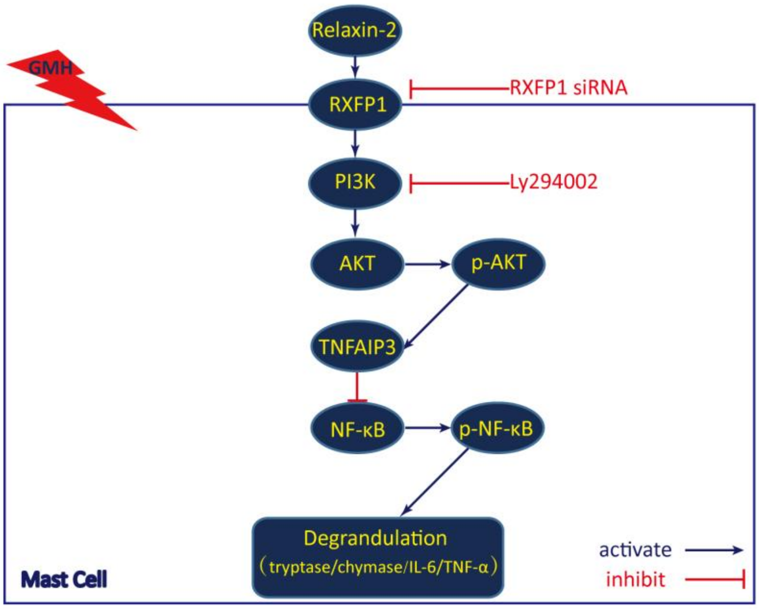

Supplementary Figure 1. Proposed mechanism.

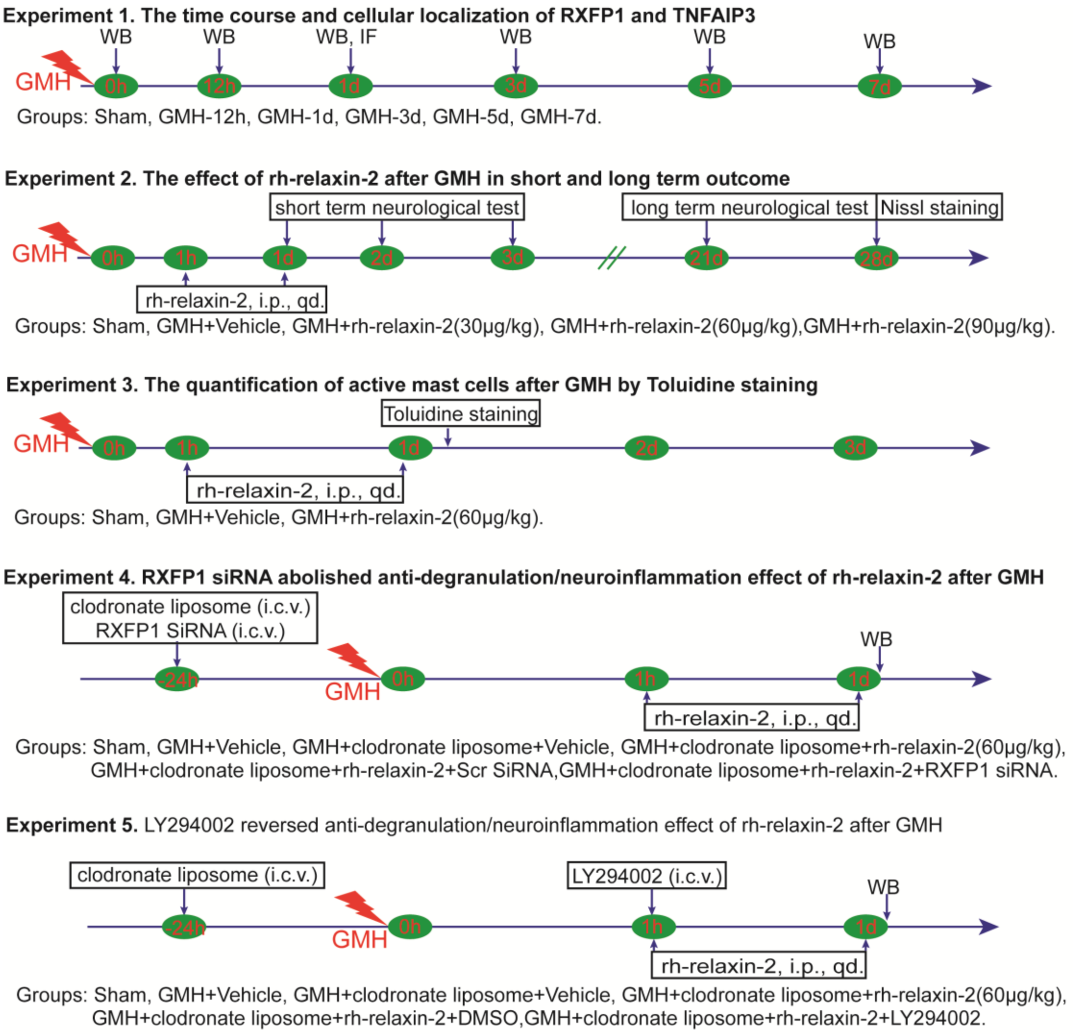

Supplementary Figure 2. Experimental design.

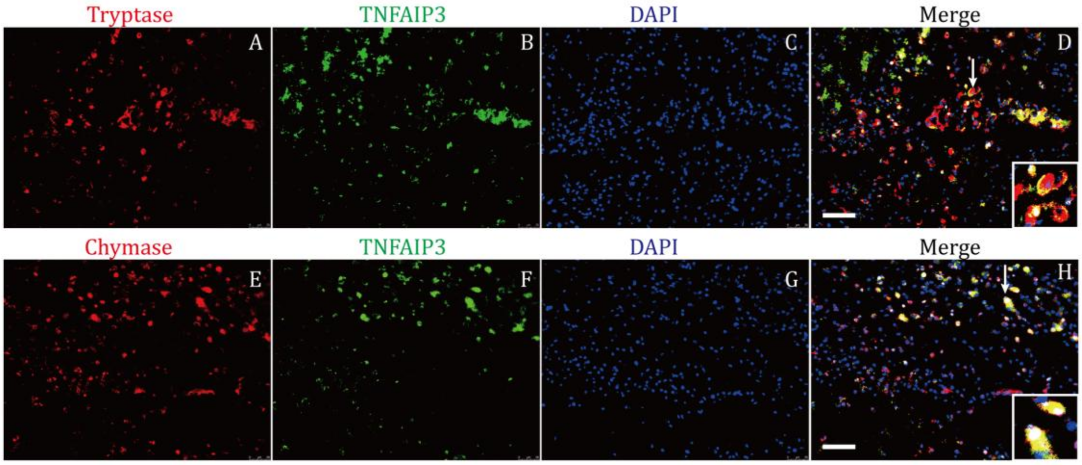

Supplementary Figure 3. The cellular localization of TNFAIP3 in the perihematoma area of brains. Representative of double immunofluorescence staining showed that TNFAIP3 (B and F) was expressed on mast cells marked with Tryptase (A) and Chymase (E) on the first day after GMH. n=6. Scale bar=50μm.

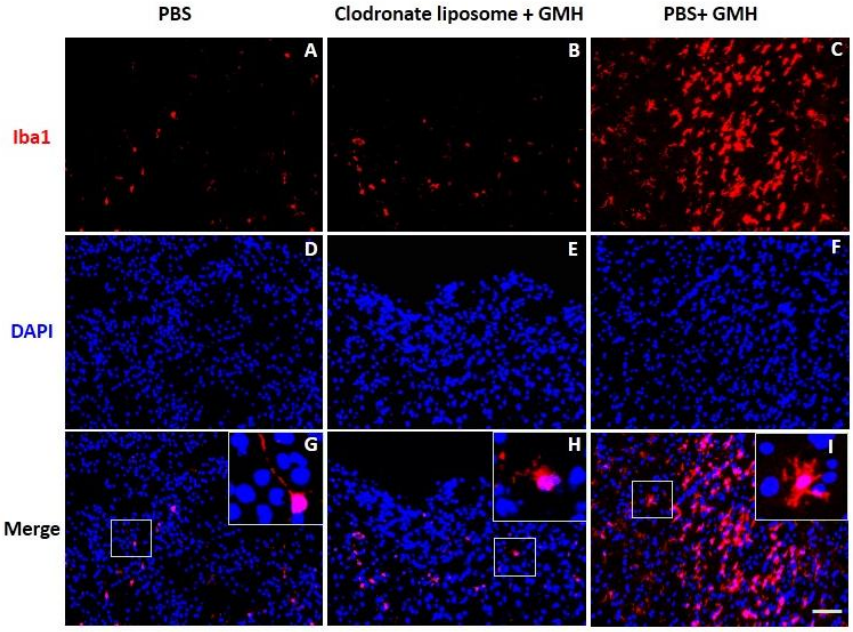

Supplementary Figure 4. Clodronate liposome inhibits the response of microglia in GMH rat model. Representative of immunofluorescence staining showed the expression of Iba1 in clodronate liposome + GMH group (B, H) was inhibited compare to PBS + GMH group (C, I) on the first day after GMH. n=3, scale bar=50μm.

Groups	numbers
**Experiment 1 (time course)**	
naive	6
GMH(12h,1d,3d,5d,7d)	30
**Experiment 1 (IF)**	
sham	6
GMH+vehicle	6
GMH+rh-relaxin-2(60μg/kg)	6
**Experiment 2 (outcome study)**	
sham	7+3
GMH+vehicle	7+3
GMH+rh-relaxin-2(30μg/kg)	7
GMH+rh-relaxin-2(60μg/kg)	7+3
GMH+rh-relaxin-2(90μg/kg)	7
**Experiment 3 (Toluidine Staining)**	
sham	6
GMH+vehicle	6
GMH+rh-relaxin-2(60μg/kg)	6
**Experiment 4 (mechanism)**	
Sham	6
GMH+Vehicle	6
GMH+clodronate liposome+Vehicle	6
GMH+clodronate liposome+rh-relaxin-2(60μg/kg)	6
GMH+clodronate liposome+rh-relaxin-2+Scr SiRNA	6
GMH+clodronate liposome+rh-relaxin-2+RXFP1 siRNA	6
GMH+clodronate liposome+rh-relaxin-2+DMSO	6
GMH+clodronate liposome+rh-relaxin-2+LY294002	6
**Experiment 5 (clodronate liposome function)**	
PBS	3
clodronate liposome + GMH	3
PBS + GMH	3
**Total**	173

Supplementary Table 1. Animal use in each experimental group.
